# Towards map-based cloning of *FB_Mfu10*: identification of a receptor-like kinase candidate gene underlying the *Malus fusca* fire blight resistance locus on linkage group 10

**DOI:** 10.1007/s11032-018-0863-5

**Published:** 2018-08-06

**Authors:** Ofere Francis Emeriewen, Klaus Richter, Stefano Piazza, Diego Micheletti, Giovanni A. L. Broggini, Thomas Berner, Jens Keilwagen, Magda-Viola Hanke, Mickael Malnoy, Andreas Peil

**Affiliations:** 1 Julius Kühn-Institut (JKI), Federal Research Centre for Cultivated Plants, Institute for Breeding Research on Fruit Crops, Pillnitzer Platz 3a, 01326 Dresden, Germany; 2 Julius Kühn-Institut (JKI), Federal Research Centre for Cultivated Plants, Institute for Resistance Research and Stress Tolerance, Erwin-Baur-Str. 27, 06484 Quedlinburg, Germany; 3 Research and Innovation Centre, Fondazione Edmund Mach (FEM), Via E. Mach 1, 38010 San Michele all ‘Adige, Italy; 4 Swiss Federal Institute of Technology, Molecular Plant Breeding, Zurich, Switzerland; 5 Julius Kühn-Institut (JKI), Federal Research Centre for Cultivated Plants, Institute for Biosafety in Plant Biotechnology, Erwin-Baur-Str. 27, 06484 Quedlinburg, Germany

**Keywords:** *Erwinia amylovora*, Recombinants, Molecular markers, BACs, Candidate gene

## Abstract

Breeding for resistance against the destructive fire blight disease of apples is the most sustainable strategy to control the menace of this disease, and has become increasingly important in European apple breeding programs. Since most cultivars are susceptible, wild accessions have been explored for resistance with quantitative trait loci detected in a few wild species. Fire blight resistance of *Malus fusca* was described following phenotypic evaluations with a C-type strain of *Erwinia amylovora*, Ea222_JKI, and the detection of a major QTL on chromosome 10 (*Mfu10*) of this crabapple. The stability of the resistance of *M*. *fusca* and *Mfu10* has been evaluated using two other strains, the highly aggressive Canadian S-type strain—Ea3049, and the avrRpt2_EA_ mutant—ZYRKD3-1, both of which overcome the resistance of *Malus* ×*robusta* 5, a wild species accession with an already described fire blight resistance gene. To pave the way for positional cloning of the underlying fire blight resistance gene of *M. fusca*, we have fine mapped the QTL region on linkage group 10 using 1888 individuals and 23 newly developed molecular markers, thus delimiting the interval of interest to 0.33 cM between markers FR39G5T7xT7y/FR24N24RP and FRMf7358424/FR46H22. Tightly linked SSR markers are suitable for marker-assisted selection in breeding programs. Furthermore, a bacterial artificial chromosome (BAC) clone spanning *FB_Mfu10* region was isolated and sequenced. One putative fire blight resistance candidate gene of *M. fusca* was predicted on the sequence of BAC 46H22 within the resistance region that encodes B-lectin and serine/threonine kinase domains.

**Electronic supplementary material:**

The online version of this article (10.1007/s11032-018-0863-5) contains supplementary material, which is available to authorized users.

## Introduction

Apple (*Malus domestica* Borkh.) is one of the most economically important fruit crops worldwide. However, one of the most serious threats to apple production is fire blight disease, caused by the bacterial pathogen, *Erwinia amylovora* (Burrill) (Winslow et al. 1920). Fire blight is considered to be the most dreadful bacterial disease affecting pome fruit production. *E. amylovora* possesses the type III secretion system (T3SS) located on the so-called pathogenicity island (PAI) which is encoded by a cluster of hypersensitive response and pathogenicity (*hrp*) genes (Khan et al. 2012). The T3SS deposits effector proteins into the host and enables the pathogen to cause disease in susceptible host plants. Primary infection occurs during flowering and depending on the degree of susceptibility of a cultivar, could spread within the entire tree in a single growing season. Fire blight has proven over the years to be very difficult to manage. Some preventive measures employed by pome fruit growers include the use of antibiotics and antagonists. Rigid pruning of infected parts of a tree or the complete eradication of affected trees are curative measures. Nevertheless, these control methods are not sustainable and cannot totally mitigate the disease in a season favorable to *E*. *amylovora*.

The use of host plant resistance is desirable and could prove to be a reliable strategy for the sustainable management of fire blight. Since 2005, several fire blight resistance quantitative trait loci (QTLs) have been described in *Malus* (Calenge et al. 2005; Khan et al. 2006; Peil et al. 2007; Durel et al. 2009; Le Roux et al. 2010; Emeriewen et al. 2014a; Emeriewen et al. 2017a; van de Weg et al. 2018). The strongest QTLs reported in wild apple species are located on linkage group 3 (LG3) of *Malus* ×*robusta* 5 (Mr5; Peil et al. 2007) and LG10 of *Malus fusca* (Emeriewen et al. 2014b), explaining 80 and 66% respectively of the phenotypic variation to the same pathogen strain Ea222_JKI. However, resistance to fire blight has been shown to be strain-specific (Norelli and Aldwinkle 1986; Paulin and Lespinasse 1990; Peil et al. 2011; Vogt et al. 2013; Wöhner et al. 2014).

Bacterial effectors, such as the type III effector protein avrRpt2_EA_ (Vogt et al. 2013) play critical roles via the type III secretion system (T3SS) in regulating plant susceptibility and circulating the pathogen (Khan et al. 2012). A single nucleotide mutation in the *avrRpt2*_*EA*_ effector of *E*. *amylovora* was responsible for a switch to virulence on Mr5 (Vogt et al. 2013). Furthermore, the loss of the avrRpt2_EA_ effector was proven to cause susceptibility in Mr5 (Vogt et al. 2013). Recently, an *eop1* mutant of *E. amylovora* led to significantly higher disease incidences in *M. floribunda* 821 (Mf821) and the ornamental cultivar ‘Evereste,’ suggesting at least a partial breakdown of their fire blight resistances and hence indicating a new gene-for-gene relationship in *Malus* (Wöhner et al. 2017). Although the fire blight resistance QTL of Mr5 on LG3 is broken-down by avrRpt2_EA_ S-type strains (Peil et al. 2011, Vogt et al. 2013), the underlying resistance gene was described by Fahrentrapp et al. (2013). The gene, *FB_MR5*, an NBS-LRR gene, belonging to class 2 of plant R genes (Kruijt et al. 2005), was hypothesized to monitor RIN4 from Mr5, a homolog to RIN4 of *Arabidopsis thaliana* (Fahrentrapp et al. 2013). Furthermore, *FB_MR5* is the first, and currently the only functionally characterized fire blight resistance gene of apple (Broggini et al. 2014). Additionally, the proposed function of *FB*_*MR5* in a cisgenic apple was demonstrated by Kost et al. (2015).

Taking the strain specificity of fire blight resistance into account, it is important for breeding to aim at pyramiding different fire blight resistance QTLs for durable resistance (Emeriewen et al. 2015). Therefore, the identification of fire blight resistance donors with different mechanisms and the subsequent isolation and functional verification of the underlying genes is necessary. Till date, the *M. fusca* accession MAL0045 and the corresponding fire blight resistance QTL on linkage group 10 (*Mfu10*; Emeriewen et al. 2014b) have not been overcome by any strain of *E. amylovora* tested (Emeriewen et al. 2015, 2017b; Wöhner et al. 2017). Therefore, the *M. fusca* fire blight resistance QTL on LG10 (*Mfu10*) can play a critical role in pyramided resistance for durability especially since it possesses a different resistance mechanism from the Mr5 QTL on LG3 and the QTLs from ‘Evereste’ and Mf821 on LG12 (Emeriewen et al. 2015; Wöhner et al. 2017). Here, we report the construction of a fine genetic map of the region containing *Mfu10* after enrichment with 23 tightly linked markers. Genome walking approach led to the isolation of BAC clones in the *Mfu10* putative region and the subsequent identification of a candidate gene from the assembled sequence of two clones in this region.

## Materials and methods

### Plant materials and DNA extraction

The original mapping population (ID: 05210) of 134 individuals which was derived from a cross of *M. fusca* × ‘Idared’ (Emeriewen et al. 2014b) was enlarged for the current study to a total number of 1888 individuals. Therefore, five populations (ID: 12228, 12229, 16250, 16251, and 16252) were developed from crosses between the fire blight resistant *M. fusca* accession MAL0045 and the susceptible *M. domestica* cultivar ‘Idared’ (Table 1). Besides the original mapping population where cetyltrimethylammonium bromide (CTAB) DNA extraction procedure was used (Doyle and Doyle 1987), DNA was extracted by adding a piece of leaf (4 mm diameter) into 50 μl of extraction solution (Sigma Aldrich, Hamburg, Germany) followed by heating in a thermo cycler for 10 min at 95 °C and then adding 50 μl of Extract-N-Amp plant dilution solution (Sigma Aldrich, Hamburg, Germany) before removing the leaf material. DNA was diluted 1:5 and stored in − 20 °C until required for PCR.

**Table 1:** All six populations used in this study with a total number of 1888 progeny individuals

Population name	Cross	Number of individuals	Number of recombinants in the interval from	
			CH03d11 to FR149B	FR20D to FR22A
*05210	*M. fusca* × Idared	134	17	0
12228	Idared × *M. fusca*	155	25	4
12229	*M. fusca* × Idared	978	110	17
16250	Idared × *M. fusca*	436	N/D	7
16251	*M. fusca* × Idared	171	N/D	3
16252	*M. fusca* × Idared	14	N/D	0
Total		1888	152	31

*Original mapping population from Emeriewen et al. (2014b)

*N/D* not determined

### Marker development and polymorphism tests

Since SSR markers FRM4 and FR481A mapping on chromosome 10 of *M. fusca* were the closest and most significantly linked to *Mfu10* (Emeriewen et al. 2014b), their location became the starting point for the development of other tightly linked markers. To saturate the region, markers were developed from contigs surrounding the position of both SSR markers on the pseudo-chromosome 10 of ‘Golden Delicious’ (Velasco et al. 2010). Markers were also developed from sequences of paired end short reads of *M. fusca* (http://vannocke.hrt.msu.edu/DBI-0922447/Genomic_Mfusca.html) as well as from sequenced T7 and RP ends of identified BAC (Bacterial artificial chromosome) clones and from BAC sequences. In general, available sequences were preferably scanned for SSR motifs and flanking primers were developed using Primer 3 (Rozen and Skaletsky 2000). To test for polymorphism, developed markers were first tested on both parents and a subset of six progenies of the 05210 population, three of which inherited the allele of FRM4 in coupling with resistance (156 bp) and the other three inheriting the allele in repulsion to resistance (166 bp) as reported in Emeriewen et al. (2014b). Polymorphism was tested according to Schuelke (2000). When markers were polymorphic and segregating properly among the six selected individuals, some forward primers of the markers (Table 2) were ordered with labeled dyes depending on the Sequence Analyzer to be used for analyses to enable multiplexing of primers, i.e., either 3730XL DNA analyzer (Applied Biosystems, Vienna, Austria) or CEQ 2000XL DNA sequencer (Beckman Coulter, Germany).

**Table 2:** Molecular markers developed from contigs of pseudo-chromosome 10 of the ‘Golden Delicious’ genome

Marker name	Forward primer	Reverse primer	Resistant allele (bp)	Susceptible allele (bp)
FR19B	GCTATACAGCTACAGCAAGCAGA	GCATGGAATCTTTTTATTCCCTTA	213^a^	201
FR202	CCTCCAACAATTCACCAACC	TTGTCGCCATAGTTGCTCAG	222^a^	201
FR20C	AGTATGGGGTGACATGCAGA	CCCTCTCTCTTCCCCTCATC	162	164
FR20D	CCTTGCTTGCATTATCTCAGC	AAATGTCGGCAAGTCCACTC	123	125
FR21BB	GCTCGATCTGGTGGTGATTG	CAAGGAAAACGTGGCCATCA	220^a^	Ø
FR21Dii	GAGGTAGGGTGGGGTTGATT	ACTTTGCGCCATGTTGATAA	Ø	163^a^
FR21T-nu	AACCTCGAATGTTGCTGCTC	ATAAGGGACAGGGCATGAGG	158^a^	113
FR22A	CGGGAACAAAACCAAGAAGA	TCCAATGTTGCAAAAGCAAA	212	210
FR22Ai	GGCCATCCACTGTCTTCTGT	CGGCCTCTTGCCATATCTTA	227	235
FR24N24_RP^b^	TCTTGGCACCCAACAAAACTT	TAGGCCCAAAGAAAATGAGG	200^a^	Ø
FR342i	GCAAGCCCTGTAAATGCAAC	CAAATCAGATACATGGGCGGA	231^a^	235
FR34C2^b^	TCTAAGTGGCCCATCAGCAG	CTTCCGTGTCCCAACTCCTA	238^a^	Ø
FR39G5T7xT7y^b^	CAATAAGAGGCCGGCAATAC	GCATGTGTGTGAAAGGGTGT	478^a^	571
FR46H22^b^	GGTTCTGAGCACATTCTTCCA	TGCTAGTTCACCGTGACTGT	229^a^	Ø
FR132	GGCACAAGACTGACATGAATC	GACCACGAACTTGATGAGCA	242/244	210^a^
FR210	TATTTCTGTGCCCGCCTTCT	GCTTCAAGGGCACGATGT	191^a^	185
FRG5342^c^	CCTTGGCTCTGATACCCAACT	GGGTTCTCTCGTTCATCCAA	211^a^	229
FRMf7334158i^d^	CGTACGATTGGGTAGGCAGT	CACACTTCCATCGCTGATTC	Ø	198^a^
FRMf7358424^d^	CAGTTGGTGCATGTCGAAAA	TGCTGTATCATTGGTGCTCA	222 (G)^a^	222 (A)
ISY213	CCATCAGGTACTGCAAAGCA	AGTACCAAGCAACTGATGCAA	233^a^	238
OFE	AAGGCCGAGGTTGGTAAGAC	TGGTTGCATTCACTTTGAGG	185^a^	191
V_BORE	AGTAAACTCGATCCCCACGA	CCCTGTGCTCCTGACATACC	197/283	190^a^
YO_FC1^c^	CCGTTGCCTTTACGAACACT	GAGCAGAGCAGAGAGAGTGGA	248^a^	244

^a^Size with elongated primer (according to Schuelke 2000)

^b^Developed from BAC-ends

^c^Developed from sequences of BAC clones

^d^Developed from sequences of paired end reads of *M*. *fusca*

Ø, null allele; *bp,* base pair

### Sequencing of marker fragments to detect SNPs

Markers possessing alleles not polymorphic in the population were used to sequence both parents and selected progenies including recombinants (resistant and susceptible) in an attempt to identify SNP sites segregating among the two classes of progenies. Amplified PCR products were purified using MSB® Spin PCRapace KIT (Invitek GmbH, Berlin, Germany) according to the manufacturer’s instructions. Sequencing of amplified products was performed by Eurofins MWG Operon (Ebersberg, Germany). Sequences were analyzed using BioEdit program.

### Marker application and identification of recombinant individuals

Firstly, the 12228 and 12229 populations, 1133 individuals in total, were screened with the two SSR markers (CH03d11 and FR149B) bracketing the QTL and then with the two SSRs, FRM4 and FR481A, tightly linked to resistance within this interval in order to identify individuals showing recombination events in the 15.79 cM interval (Emeriewen et al. 2014b). Application of these markers was performed using a PCR approach in a volume of 10.2 μl comprising 1 × GeneAmp PCR buffer II (Applied Biosystems, Monza, Italy), 2.4 mM MgCl_2_, 0.05 mM dNTPs, 0.75 U AmpliTaq Gold DNA polymerase (GeneAmp, Applied Biosystems, Monza, Italy), 2 mM SSR primer mix (forward and reverse primers) with the following profile: 94 °C for 10 min, followed by 30 cycles of 94 °C for 30 s, 60 °C for 1 min and 72 °C for 1 min, and an extension of 72 °C for 5 min. PCR fragments were analyzed on a 3730XL DNA analyzer (Applied Biosystems, Vienna, Austria). Once recombinants from the 12228 and 12229 populations were identified and their linkage to resistance determined, the interesting ones were then genotyped with newly developed markers (Table 2) to more precisely determine the region of interest. For this purpose, PCR was performed using the Type-It Kit (Qiagen, Hilden, Germany) in a 10 μl volume with the following conditions: 95 °C for 5 min, followed by 30 cycles of 95 °C for 1 min, 60 °C for 1 min 30 s, and 72 °C for 30 s, and a final extension for 30 min at 60 °C. PCR fragments were then analyzed on a CEQ 2000XL DNA sequencer (Beckman Coulter, Germany). Sample preparations for the 3730XL DNA analyzer and the CEQ 2000XL DNA sequencer are as previously reported in Emeriewen et al. (2014b). Afterwards, the other populations were screened as described above with newly developed markers to determine informative recombinants in the shortened region of interest.

### Fire blight phenotypic evaluation of recombinant individuals

For phenotypic evaluation of recombinant individuals, up to 10 replicates of each recombinant individual were grafted onto M9 rootstock and grown in the greenhouse before being transferred to the quarantine greenhouse where artificial *E*. *amylovora* inoculations were carried out with the parents *M. fusca* and ‘Idared’ as controls. Inoculation was performed on plants with a minimum length of 25 cm by incising the two youngest leaves with a pair of scissors dipped into *E*. *amylovora* inoculum of strain Ea222_JKI at a concentration of 10^9^ cfu/ml (Peil et al. 2007; Emeriewen et al. 2014b). Greenhouse conditions were 25–27 °C (day), 20 °C (night), and 85% air humidity. Shoot length and lesion length of replicates of each genotype were measured 28 days post inoculation (dpi), transformed into percentage lesion length per shoot (PLL) as initially described by Peil et al. (2007), and then averaged.

### Fine mapping of the fire blight resistance locus

Genotypic data of individuals were analyzed and mapped using JOINMAP 4.0 (Van Ooijen 2006). Phenotypic quantitative data (PLL) were transformed into binary marker data for genetic mapping (Durel et al. 2009). Recombinants were then arranged in ascending order of PLL. The mean and median PLLs in this order were then determined. While recombinants with PLL values lower than the mean PLL were classed as resistant, those with higher PLL were classed as susceptible. 10% of the recombinants between CH03d11 and FR149B from either side around the median PLL which were designated as being on the border between resistant and susceptible were excluded from further analyses. Phenotypic marker data were then compared with genotypic data. Once it was established that phenotypic data conformed to genotypic data of recombinant individuals, the binary marker data for non-recombinant individuals were then deduced by allocating 0 (i.e. resistant) and 1 (i.e. susceptible) to individuals possessing either resistant or susceptible alleles of tightly linked markers. Kruskal-Wallis analysis was performed to determine marker-phenotype association followed by interval mapping and multiple QTL mapping (MQM) using MAP-QTL 4.0 version (Van Ooijen and Maliepaard 1996).

### *Malus fusca* BAC library construction and isolation of clones

The *M. fusca* bacterial artificial chromosome (BAC) library was produced using high molecular weight genomic DNA prepared at Amplicon Express (Pullman, Washington, USA) as described by Tao et al. (2002). DNA was partially digested with *Hin*dIII, size selected and ligated into pCC1BAC® vector (Epicenter, Madison, USA) and transformed into DH10B *E. coli* cells. Clones were arrayed on 384-well plates with LB media and frozen. The BAC library screening was performed in two separate rounds of PCR on extracted DNA from independently grown, separately pooled BAC clones. The first round of the PCR was performed on 14 Super Pools containing all BAC clones of the *M. fusca* library. The second round of PCR was then performed on the respective matrix pools to determine plate, row and column (each position representing a single or multiple BAC clones) reacting positively to the applied marker. Markers used to screen the *M. fusca* BAC library are listed in Table S1. PCR reactions and fragment analysis for the two rounds of screening were performed as described above.

Identified BAC clones were cultured overnight at 37 °C in LB medium containing 12.5 μg/mL Chloramphenicol. Plasmid DNA was thereafter extracted from clone cultures using NucleoBond® Xtra MIDI Plasmid DNA Purification Kit (MACHEREY-NAGEL, Düren, Germany) according to the manufacturer’s protocol. Primers pCC1_RP (CTCGTATGTTGTGTGGAATTGTGAGC) and T7 (TAATACGACTCACTATAGGG) were used to sequence the ends of BACs. Sequencing of BAC-ends was performed by Microsynth AG (Balgach, Switzerland). Positions of BACs compared to the Golden Delicious Doubled Haploid genome (GDDH13) sequence (Daccord et al. 2017) were determined by blasting BAC sequences to the GDDH13 sequence. If the BLAST resulted in multiple hits, the most probable position of the BAC was chosen. De novo sequencing and assembly of BAC clones isolated in the resistance region as well as the susceptible homolog region was performed by various companies. Microsynth AG (Balgach, Switzerland) sequenced clones 46H22, 24N24, 36P10 and 70N1 using MiSeq Illumina sequencing. GATC Biotech (Cologne, Germany) and Amplicon Express (Pullman, USA) sequenced clones 62J21 and 39G5, respectively, using PacBio sequencing technique.

The raw sequencing data of the two overlapping resistant BACs, 24N24 and 46H22 were preprocessed together using Trim Galore. Subsequently, KmerGenie (version: 1.6715: default parameter) was run to determine the optimal k-value for a de Brujin assembly. Finally, CLC assembly cell was used to assemble the data into contigs. For aligning the BAC-ends to the assembled contigs, megablast with default parameters was used.

### Gene prediction and expression of transcripts of candidate gene

Obtained sequences were used to predict open reading frames (ORFs) using FGENESH with algorithms for dicot plants, namely *Arabidopsis*, *Solanum lycopersicum*, and *Vitis vinifera*. The predicted proteins were analyzed using the National Centre for Biotechnology (NCBI) Blastp program (Altschul et al. 1997, 2005) and ExPASy PROSITE (Bairoch 1991; Hulo et al. 2008) to predict their domains, families, and functional sites.

The putative fire blight resistance gene of *M. fusca* was amplified with the primer pair named MVH_FB_Mfu10 (forward: GCTAGCTGCAGAACTTGCTTGCTCA and reverse: GAGAAAGAGAAAACCGCCCCGTCT) developed on the ATG start codon and TGA stop codon, respectively, using genomic DNA and BAC DNA as templates. Additionally, the candidate gene ORF including 1537 bp of the genomic regions upstream of the ATG codon and downstream of the TGA codon, respectively, with a length of 6366 bp, was amplified using genomic DNA and BAC DNA as templates with the primer pair named FB_Mfu10_RSeq1 (forward: TTCATATCAGCCCTTTTAACCACTGCTACA and reverse: GCCATAATTAAAACCTAGCCACTATCCCGT). PCR was performed using the DreamTaq Hot Start PCR Master Mix Kit (Thermo Scientific, Berlin, Germany) in a 12 μl volume with the following conditions: 95 °C for 5 min, followed by 34 cycles of 95 °C for 1 min, 68 °C for 90 s and 72 °C for 6 min, and an extension of 72 °C for 10 min.

To ascertain the candidate gene sequence, amplicons amplified by *M. fusca*, a resistant progeny, a susceptible progeny, and the susceptible BAC clone 94B13 with the developed gene primers were sequenced. Sequencing was performed by Eurofins MWG Operon (Ebersberg, Germany) with sample preparation as initially described. Comparison of obtained sequences was done using the National Centre for Biotechnology (NCBI) Blastn function (Zhang et al. 2000).

To confirm transcription of the candidate gene, RNA was extracted from *M. fusca* in vitro seedling using InviTrap® Spin Plant RNA Mini Kit according to the manufacturer’s protocol (Stratec, Berlin, Germany). Potential DNA contamination was removed using the Invitrogen DNA-free™ Kit (Thermo Scientific, Berlin, Germany). The RevertAid First Strand cDNA Synthesis Kit (Thermo Scientific, Berlin, Germany) was used to produce cDNA according to the manufacturer’s protocol. Primer pair called FR_FB_Mfu10 (forward: AAAGCGGATATTTATTGGCACTGGTATCAC and reverse: AACGGTGTGCTTTGATTTGTCAACATAGAT) was developed to amplify parts of the ORF on cDNA.

## Results

### Increase of mapping individuals

To fine map the fire blight resistance locus of *M. fusca*, five more populations besides the original mapping population (05210) were established at different stages of this study. All six populations employed in this study with a total of 1888 individuals are shown in Table 1.

### Marker development

Initially, SSR markers FR481A and FRM4 which correlated with the highest significance to fire blight resistance mapped at 35.56 cM and 37.09 cM respectively on LG10 of *M. fusca* and were bracketed by two other SSRs, CH03d11 and FR149B (Emeriewen et al. 2014b). Therefore, to saturate the interval between CH03d11 and FR149B, 15 more polymorphic SSR markers which were developed from the corresponding pseudo-chromosome 10 of the ‘Golden Delicious’ genome have been mapped (Table 2). Additionally, two polymorphic SSRs, YO_FC1 and FRG5342 were developed from the assembled sequences of BAC clones. Similarly, four markers were developed from BAC-ends. Two other markers, FRMf7334158i and FRMf7358424, were developed from *M. fusca* sequences (http://vannocke.hrt.msu.edu/DBI-0922447/Genomic_Mfusca.html). Whereas both markers were developed from SSR motifs, only FRMf7334158i was polymorphic as an SSR marker. Therefore, to identify possible SNPs, fragments amplified with FRMf7358424 were sequenced. Hence, amplicons of *M. fusca*, ‘Idared’, three progenies inheriting the 156 bp allele of FRM4 (in coupling with resistance), and three progenies inheriting the 166 bp allele of FRM4 (in repulsion with resistance) were sequenced. Analysis of the sequences revealed a single nucleotide polymorphism (SNP) at position 57 of the amplicons segregating between both classes of progenies. While nucleotide *G* was inherited by the resistant progenies, the susceptible progenies inherited *A* from *M. fusca*. Thus, FRMf7358424 was designated as a segregating marker.

### Identification and phenotyping of recombinant individuals for fine mapping of *FB_Mfu10*

**Fig. 1:**
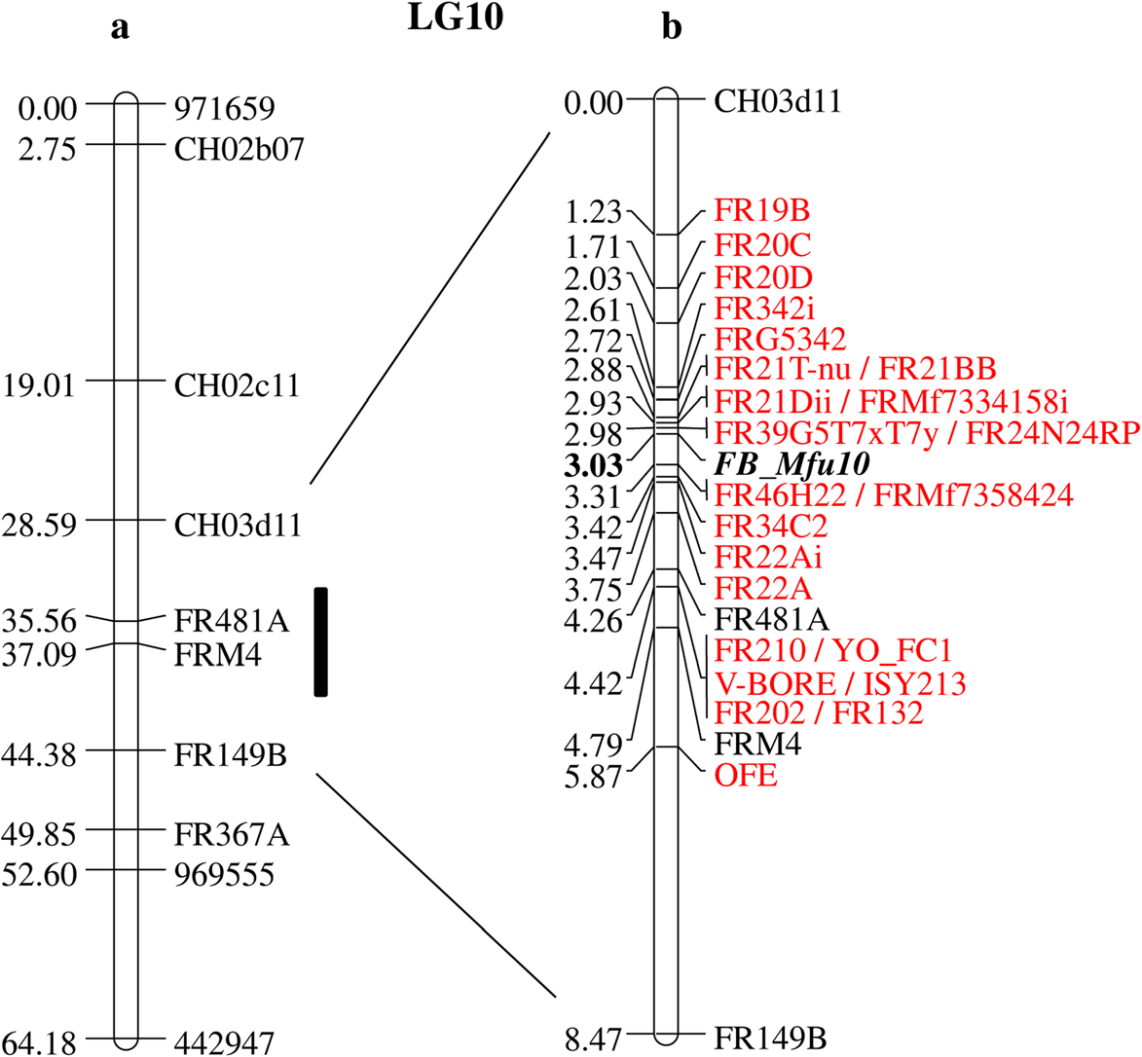
Genetic map of *M. fusca* LG10 showing (**a**) map developed with 05210 population (Emeriewen et al. 2014) with the region of interest (interval between CH03d11 and FR149B) represented with a black bar, (**b**) only the interval between CH03d11 and FR149B saturated with 23 new tightly linked molecular markers (highlighted in red) and the position of *FB_Mfu10* mapped as a single gene using all 6 populations

To identify individuals showing recombination events within the 15.79 cM interval between markers CH03d11 and FR149B (Fig. 1a) containing the fire blight resistance QTL of *M. fusca* (Emeriewen et al. 2014b), both markers were first applied on the 12228 and 12229 populations. Genotyping resulted in the identification of 25 recombinant individuals from the 12228 population and 110 from the 12229 population, in addition to the 17 recombinants already identified in 05210 population (Table 1).

In total, 152 individuals showing recombination events between CH03d11 and FR149B could be identified from the 05210, 12228, and 12229 populations of 1267 individuals. For phenotyping, individuals of 12228 and 12229 populations showing recombination events between the interval of markers FR20D and FRM4 (deemed as definitely containing *FB_Mfu10* from mapping results of Fig. 1b) were analyzed. This reduced the recombinants to be analyzed from 152 to 106 individuals. Inoculation was performed using *E*. *amylovora* isolate Ea222_JKI with the parents *M. fusca* and ‘Idared’ as controls. Results showed that whereas the resistant parent *M. fusca* showed no sign of lesion in all replicates, an average percentage lesion length (PLL) of 64% was recorded for the susceptible parent—‘Idared’. The distribution of the susceptibility/resistance level of the 12228 and 12229 phenotyped recombinants is shown is Fig. S1. A zero PLL was observed in 15 recombinant individuals whereas no 100% lesion length was recorded in all 106 recombinants. The highest PLL recorded was 83.0%. The mean PLL recorded for all 106 recombinant individuals phenotyped was 19.9% with a median of 11.5%.

**Fig. 2:**
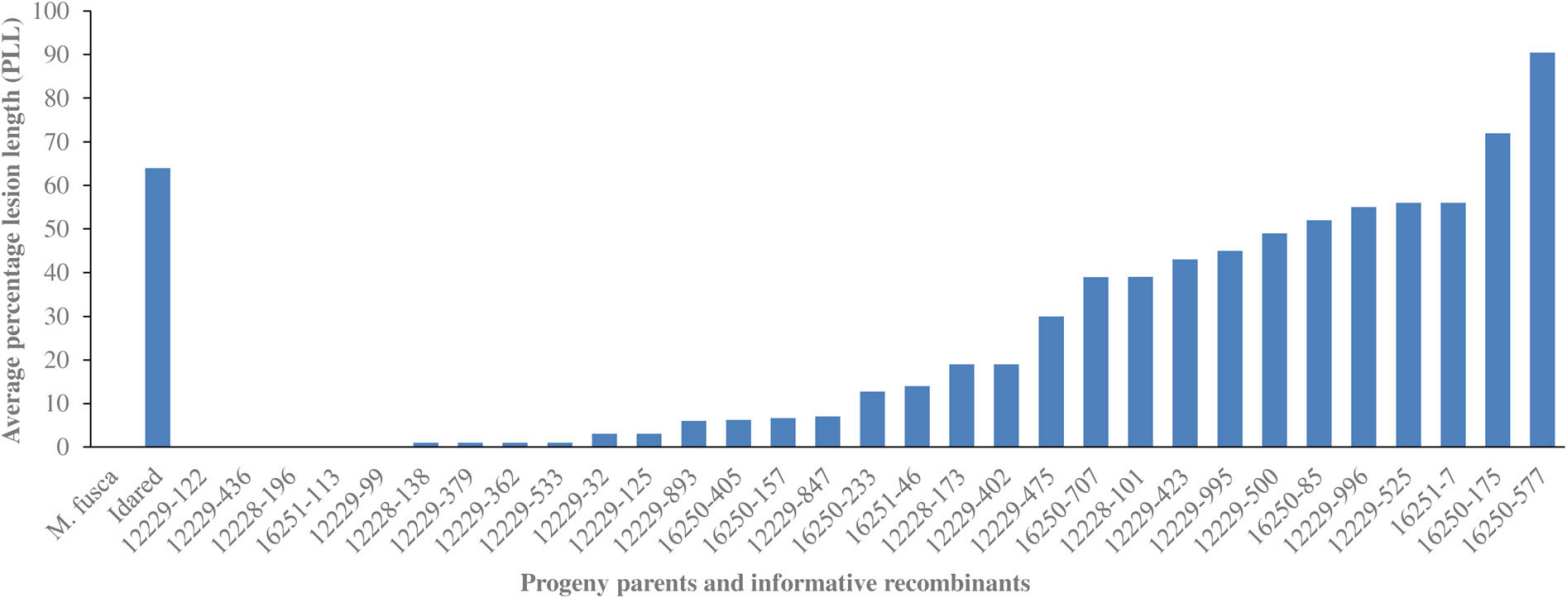
Distribution of 31 informative recombinant individuals showing their different levels of resistance/susceptibility to *E*. *amylovora*. Individuals are ordered according to percentage necrosis (PLL)

Mapping with 05210, 12228, and 12229 populations ensured that the region of interest was narrowed down to the 1.72 cM interval between markers FR20D and FR22A (Fig. 1b). To further delimit the interval carrying *FB_Mfu10*, it was then necessary to identify more recombinant individuals. Hence, three other populations (16250, 16251, and 16252) were established comprising a total of 621 individuals. Of these 621 individuals, ten showed recombination events between FR20D and FR22A. In general, from all six populations used in this study, 31 individuals showing recombination events within the interval between FR20D and FR22A were informative in determining the precise position of *FB_Mfu10*. The phenotyping results of all 31 recombinant individuals are shown in Fig. 2.

**Fig. 3:**
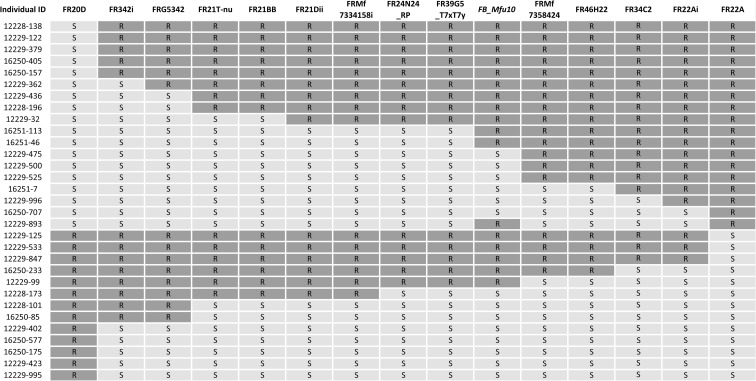
Graphical representation of *FB_Mfu10* region with 31 individuals showing recombination events between SSR markers FR20D and FR22A. *R* = resistant, *S* = susceptible. The genotype and phenotype of 12229-893 did not correspond

Fine mapping *FB_Mfu10* using the 23 genetic markers (Table 2) and the binary fire blight data finally led to the delimiting of the region to 0.33 cM (Fig. 1b) between markers FR39G5T7xT7y/FR24N24RP and FRMf7358424/FR46H22. An overview of the informative recombinants combined with their binary phenotypes is shown in Fig. 3. One recombinant (12229-893) in this region showed phenotype/genotype incongruity.

### Isolation and sequencing of BAC clones

**Fig. 4:**
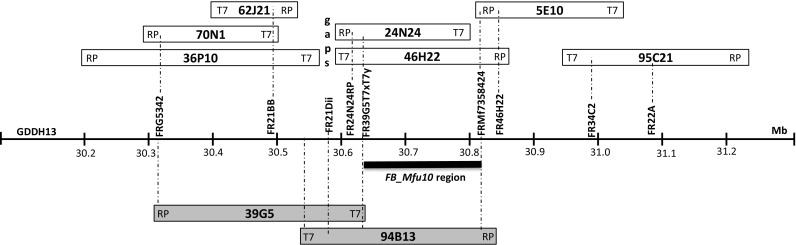
Schematic representation of *FB_Mfu10* region with BAC clones from the fire blight resistant and susceptible (in gray) regions of *M. fusca*. Dotted lines show sequence alignment/indicate markers used to detect clones. Marker and clone positions are also estimated on the Golden Delicious doubled haploid genome (GDDH13)

To identify BAC clones in the region of interest containing *FB*_*Mfu10*, the constructed *M. fusca* BAC library, which represents a 6.6 times coverage of the haploid apple genome, was screened with markers tightly linked to the fire blight resistance locus of *M. fusca*. Resistant and susceptible clones and markers used to detect them are presented in Table S1. Resistance and susceptibility were determined by the possession of resistant or susceptible alleles of the corresponding molecular marker. Some markers detected the same clones due to their mapping proximity. Sequences of BAC-ends were aligned to the Golden Delicious doubled haploid genome (GDDH13; Daccord et al. 2017) to ascertain the orientation of the clones. The positions of clones following alignment on GDDH13 are presented in Table S2. The position of BAC clones around the resistant and susceptible fire blight loci on LG10 in relation to the GDDH13 sequence is given in Fig. 4. With markers FR39G5T7xT7y/FR24N24RP and FRMf7358424/FR46H22, BAC clone 46H22 was identified spanning the fire blight resistance region. On the other hand, with markers FR39G5T7xT7y and FRMf7358424, a susceptible BAC clone (94B13) spanning the homolog region was identified (Fig. 4). Markers FR24N24RP and FR46H22 failed to detect 94B13 since both markers possess null alleles for susceptibility.

Sequencing and assembly of BAC clones 46H22 and 24N24 as well as marker data showed that 46H22 contains the entire sequence of clone 24N24. An assembly with sequence data of both clones comprised of 45 contigs (216 Kbp in total) with 88 Kbp and 205 bp being the longest and shortest contigs, respectively. The orientation of BAC contigs was in conformity with the orientation of BAC-end sequences blasted against the GDDH13 genome (Fig. 4).

### Prediction of candidate gene

**Fig. 5:**
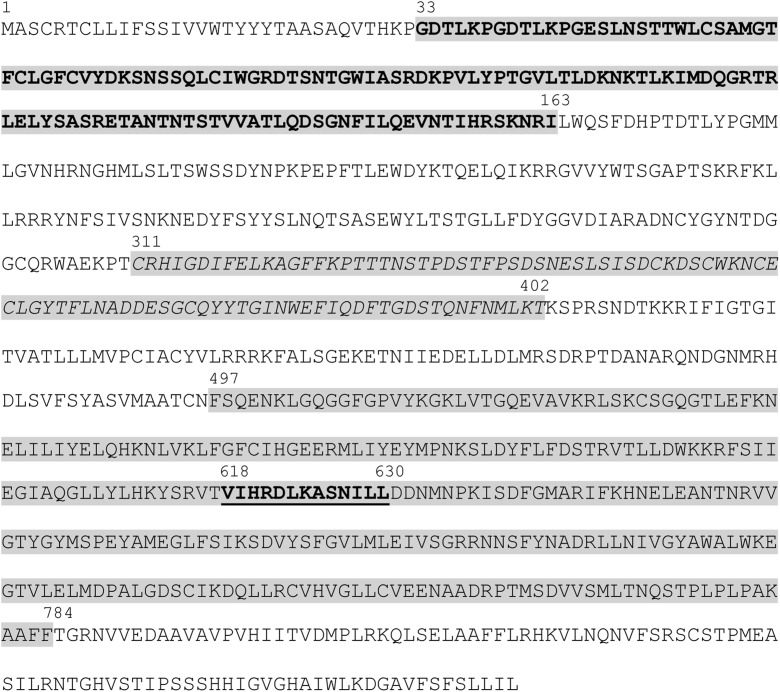
Deduced amino acid sequence of the putative fire blight resistance candidate gene of *M. fusca* showing the conserved domains as annotated using ExPASy PROSITE. 33–163, bulb-type lectin domain profile (grey background, bold); 311–402, PAN/Apple domain profile (grey background, italics); 497–784, protein kinase domain profile (grey background, upright); 618–630, serine/threonine protein kinases active-site signature (grey background, bold underlined)

Gene prediction analyses with derived sequences of 46H22/24N24 using FGENESH with algorithms for dicot plants, namely *Arabidopsis*, *Solanum lycopersicum* and *Vitis vinifera* predicted one putative gene. Analyses of the predicted protein (Fig. 5) indicated the bulb-type mannose-specific lectin (B-lectin) and a catalytic domain of the serine/threonine and interleukin-1 receptor associated kinases (STKc_IRAK), with highest identity (91%) to G-type lectin S-receptor-like serine/threonine-protein kinase of *Pyrus* × *bretschneideri*. Further analysis of the protein on ExPASy PROSITE confirmed the presence of the B-lectin domain, a serine/threonine protein kinase signature, and revealed a PAN/apple domain profile (Fig. 5). No other ORFs showing homology with resistance genes were predicted on the assembled sequences of 46H22/24N24.

### Resequencing of putative candidate gene and determination of transcript

The ORF including 1537 bp upstream and downstream, respectively (6366 bp), was amplified only on 46H22 and 24N24 BAC clones, resistant progeny, and *M. fusca* with primer pair FB_Mfu10_RSeq1. The amplified amplicon was fully resequenced and showed 100% identity with the 46H22/24N24 sequence. The ORF (3555 bp) was amplified with primer pair MVH_FB_Mfu10 in *M. fusca*, resistant progeny, susceptible progeny, BAC clones 46H22, 24N24 as well as BAC clone 94B13, but not in the susceptible parent ‘Idared’. Sequencing of the amplicon from the susceptible BAC clone 94B13 and a susceptible progeny resulted in eight base pairs difference in sequence length of the putative gene and a resultant protein sequence with 28 amino acids difference upstream. Furthermore, part of the ORF (1092 bp) was amplified on cDNA of *M. fusca* with primer pair FR_FB_Mfu10.

## Discussion

We report the identification of a putative fire blight resistance candidate gene of *M. fusca* encoding protein kinase domains known to confer resistance to plant diseases. *FB_Mfu10* was predicted on the sequence of BAC clone 46H22 which was found to span the *M. fusca* fire blight resistance region where a resistance QTL was previously reported (Emeriewen et al. 2014b). The identification of the putative candidate gene in this region was achieved following substantial increase of the initial mapping population of 134 individuals (Emeriewen et al. 2014b) with 1754 new individuals (Table 1) and mapping of 23 tightly linked molecular markers which allowed for the identification of informative recombinant individuals whose subsequent phenotypic evaluation allowed for the delimiting of the initially reported fire blight region from a 15.79 cM interval (Fig. 1a; Emeriewen et al. 2014b) to 0.33 cM (Fig. 1b). Only 31 recombinants determined the precise position of *FB_Mfu10*, however, all recombinant individuals identified in this study offered integral information from phenotyping results (Fig. 2 and S1). Despite applying the method of Durel et al. (2009) to reliably distinguish between resistant and susceptible recombinants for the transformation of binary data, i.e., the elimination of 10% of recombinants with PLL each below and above the median PLL, one individual (12229-893) in the region still showed phenotype/genotype incongruity. Such phenotype/genotype incongruity was also shown by Fahrentrapp et al. (2013) when identifying the candidate gene for fire blight resistance in *M.* ×*robusta* 5. The subsequent isolation of *FB_MR5*, the first functional fire blight resistance gene (Broggini et al. 2014), demonstrates that this approach could still be successful even with an individual showing phenotype/genotype incongruity.

Furthermore, the saturation of the fire blight region with tightly linked molecular markers such as SSRs is also important for marker-assisted selection/breeding (MAS; MAB) of pre-breeding materials prior to the introgression of this trait into related cultivars to minimize genetic drag. The initially reported interval of 15.79 cM contained only 2 SSRs (FRM4 and FR481A) bracketed by two other SSRs, CH03d11, and FR149B (Emeriewen et al. 2014b). Of the 23 newly developed molecular markers, one marker, OFE, mapped downstream of FRM4 (i.e., between FRM4 and FR149B) with six markers mapping between FRM4 and FR481A and 16 markers mapped upstream of FR481A. Importantly, with single gene/trait mapping using transformed binary data of recombinant individuals (Durel et al. 2009); *FB_Mfu10* was situated in a 0.33 cM interval between FR39G5T7xT7y/FR24N24RP and FRMf7358424/FR46H22 (Fig. 3). In similar studies, Fahrentrapp et al. (2013) showed that markers FEM14/FEM47 and rp16k15 flanked the fire blight resistance locus of Mr5 within a 0.24 cM interval while Parravicini et al. (2011) reported a distance of 0.18 cM between markers ChfbE01 and ChfbE08 flanking the resistance QTL of ‘Evereste’ with another marker, ChfbE02-7, co-localizing with the locus. In this study, no marker was found to co-segregate with *FB_Mfu10*.

### Identification of clones and physical characterization of *FB*_*Mfu10* region

The direct screening of the *M. fusca* BAC library DNA Pool with tightly linked markers is a different approach to other chromosome landing studies in *Malus* which were hybridization-based (Patocchi et al. 1999; Galli et al. 2010; Parravicini et al. 2011; Fahrentrapp et al. 2013). Although several resistant and susceptible BAC clones were identified using this PCR-based approach (Fig. 4; Table S1), not all could be well defined due to unreliable BAC-end sequences or poor sequence alignments. Nevertheless, the clones presented in Fig. 4 were sufficient for the physical characterization of *FB_Mfu10* region and its susceptible homolog, and it was possible to confirm BAC clones 46H22 and 94B13 as spanning the resistant region and susceptible homolog region, respectively. Previous fire blight genome walking approaches in Mr5 (Fahrentrapp et al. 2013) and ‘Evereste’ (Parravicini et al. 2011) also found single BAC clones spanning the respective regions.

In general, alignment of the sequences of the BAC clones and BAC-ends to the *M. domestica* doubled haploid genome (GDDH13, Daccord et al. 2017) was essential in confirming the orientation of the clones as well as characterizing the *FB_Mfu10* regions. The initial ‘Golden Delicious’ genome (Velasco et al. 2010), though useful especially in the development of new tightly linked molecular markers, was not accurate in BACs orientation due to large sequence gaps found in this genome as well as the overestimation of the distance and sizes in this region. For example, the *M. fusca* genetic region from markers FR20D to FR22A (interval of 1.72 cM) which definitely contains the fire blight resistance locus, corresponds to at least 1.2 Mbp on the ‘Golden Delicious’ genome (Velasco et al. 2010), but only about 700 Kbp on GDDH13 (Daccord et al. 2017). Although the reads of two overlapping resistant BACs 46H22 and 24N24 were used for assembly, a single contig sequence could not be achieved. Fahrentrapp et al. (2013) reported four contigs for BAC 16k15 in the fire blight region of Mr5 with an insert length of 162 Kb after pyro-sequencing. These authors were unable to still close the gaps even after Sanger sequencing and resequencing of clone 16k15 with Illumina HiSeq 2000.

### A candidate gene encoding receptor-like kinase protein

With the sequence of 46H22/24N24, a single candidate gene was predicted with protein comprising 880 amino acids that encodes a receptor-like kinase protein including a bulb-type mannose-specific binding lectin (B-lectin) domain and a catalytic domain of the serine/threonine kinases and interleukin-1 receptor-associated kinases (STKc_IRAK). Further analyses of the amino acids also revealed the PAN/APPLE-like domain (Fig. 5), predicted to bind proteins or carbohydrates. The first region, the B-lectin domain, is associated with protein-carbohydrate interactions. Upon attack, plants perceive the structures of the invading microbial organism using cell-surface and intracellular immune receptors such as lectins and proteins with one or more lectin domains (Lannoo and Van Damme 2014). Since it is established that *E*. *amylovora* requires the exopolysaccharide amylovoran (*ams*) among others for pathogenicity (Oh and Beer 2005), it is probable that the B-lectin domain of this candidate gene interacts with *ams*. STKc_IRAK is the serine/threonine signature characteristic of class 5 genes conferring resistance to bacterial diseases (Kruijt et al. 2005). The domains of the putative fire blight resistance candidate gene of *M. fusca* are the same as the domains of *Pi-d2* gene of rice (B-lectin and an intracellular serine-threonine kinase) which confers gene-for-gene resistance to rice blast caused by the fungal pathogen, *Magnaporthe grisea* (Chen et al. 2006). Similarly, receptor-like kinase genes have been shown to confer resistance to bacterial blight in rice caused by *Xanthomonas oryzae* pv. *oryzae* (Sun et al. 2004; Xiang et al. 2006), and more notably the protein kinase gene (*Pto* gene) conferring resistance to bacterial speck in tomato caused by *Pseudomonas syringae* pv. tomato (Martin et al. 1993). In apple, serine/threonine kinase genes were proposed by Parravicini et al. (2011) in the fire blight resistance region of ‘Evereste.’ The most probable candidate gene of seven identified was MdE-EaK7 with 381 amino acids and possessed an ATP-binding signature. Comparison of the amino acid sequence deduced for the putative fire blight resistance gene of *M*. *fusca* and those of MdE-EaK7 and the *Pto* gene uncovered similarities of 34 and 39%, respectively (results not shown). In contrast to Parravicini et al. (2011), only one candidate gene was found by Fahrentrapp et al. (2013) in Mr5 and in our study in the corresponding BACs. Fahrentrapp et al. (2013) argued that the existence of a single resistance gene not embedded in a cluster of paralogs could be as a result of the lack of a long-term co-evolution between *Malus* species and *E. amylovora*.

The gene prediction with the amplicon sequence of the homolog from 94B13 comprised a similar protein with a significant 28 amino acids difference upstream. A single amino acid difference was shown to distinguish resistance from susceptible alleles in the *Pi-d2* gene (Chen et al. 2006) and virulence from avirulence in the *avrRpt2*_*EA*_ effector of *E*. *amylovora* (Vogt et al. 2013). Therefore, the observed differences between the two homolog regions in the current study are enough to distinguish resistance from susceptibility.

## Concluding remarks

The results presented here are of significance given that they provide the first step towards the map-based cloning of *FB_Mfu10*. The putative fire blight resistance gene of *M. fusca* is of importance in breeding as this crabapple has proven to be resistant to all strains of *E. amylovora* tested till date, including the resistant-breaking strains of *FB_MR5* from *M*. *×robusta* 5 as well as a mutant strain causing disease incidence in ‘Evereste’ and Mf821. It is therefore imperative to prove the function of the proposed candidate gene in complementing studies. When validated, *FB_Mfu10* will allow for the breeding of new cultivars as well as the development of cisgenic cultivars with pyramided fire blight resistance genes in order to establish more durable resistance.

## Electronic supplementary material

Figure S1 Distribution of 12228 and 12229 recombinant individuals showing their different levels of resistance/susceptibility to *E*. *amylovora*. Individuals are ordered according to percentage necrosis (PLL). (PDF 933 kb)

Table S1 BAC clones detected with molecular markers covering the region of interest (DOCX 14 kb)

Table S2 Positions of BAC clones on the Golden Delicious doubled haploid (GDDH13) genome (DOCX 12 kb)
