# Supplementary figures and images for: Towards map-based cloning of FB_Mfu10: identification of a receptor-like kinase candidate gene underlying the Malus fusca fire blight resistance locus on linkage group 10
Source: Mol Breed. 2018 Aug 6;38(8):106. doi: 10.1007/s11032-018-0863-5 (PMC6096517; doi:10.1007/s11032-018-0863-5)

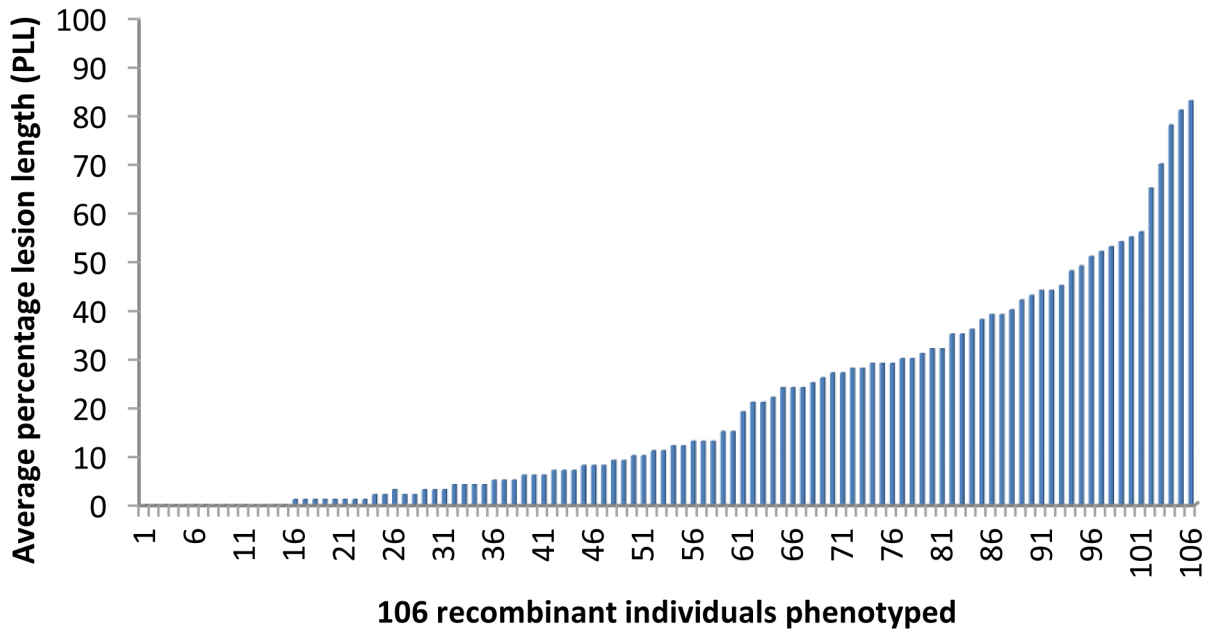

Supplement: Supplementary file 1 — Distribution of 12228 and 12229 recombinant individuals showing their different levels of resistance/susceptibility to E. amylovora. Individuals are ordered according to percentage necrosis (PLL). (PDF 933 kb) [file 11032_2018_863_MOESM1_ESM.pdf]
