# Supplementary material for: Towards map-based cloning of FB_Mfu10: identification of a receptor-like kinase candidate gene underlying the Malus fusca fire blight resistance locus on linkage group 10
Source: Mol Breed. 2018 Aug 6;38(8):106. doi: 10.1007/s11032-018-0863-5 (PMC6096517; doi:10.1007/s11032-018-0863-5)
Supplement: Supplementary file 2 — BAC clones detected with molecular markers covering the region of interest (DOCX 14 kb) [file 11032_2018_863_MOESM2_ESM.docx]

**Table S1**. BAC clones detected with molecular markers covering the region of interest

| Markers | Map Position (cM) | No. of clones | Resistant clones | Susceptible clones |
| --- | --- | --- | --- | --- |
| FR342i | 2.61 | 3 | 70N1, 36P10 | 95A10^†^ |
| FRG5342 | 2.72 | 3 | 70N1, 36P10 | 95A10^†^ |
| FR21T-nu | 2.88 | 3 | 62J21, 70N1, 36P10 | N/D |
| FR21BB | 2.88 | 3 | 62J21, 70N1, 36P10 | Ø |
| FR21Dii | 2.93 | 1 | Ø | 23B13 |
| FRMf7334158i | 2.93 | 2 | Ø | 39G5, 68P7 |
| FR24N24_RP | 2.98 | 2 | 24N24, 46H22 | Ø |
| FR39G5T7xT7y | 2.98 | 6 | 24N24, 46H22 | 94B13, 23B13^†^, 39G5, 68P7^†^ |
| FRMf7358424 | 3.31 | 3 | 46H22, 5E10, 34C2^†^ | N/D |
| FR46H22 | 3.31 | 3 | 46H22, 5E10, 34C2 | Ø |
| FR34C2 | 3.42 | 3 | 95C21, 87N9, 60H12 | Ø |
| FR22Ai | 3.47 | 3 | 95C21, 87N9, 60H12 | N/D |
| FR22A | 3.75 | 3 | 95C21, 87N9^†^, 60H12^†^ | N/D |

Ø = null alleles for either resistance or susceptibility hence it was impossible to detect resistant and susceptible BAC clones.

N/D = not determined.

^†^ Clones not characterized further.

All markers used for the detection of clones map within an interval of 1.14 cM (Centimorgan). Some markers detected the same clones due to mapping proximity.
