# Supplementary material for: Towards map-based cloning of FB_Mfu10: identification of a receptor-like kinase candidate gene underlying the Malus fusca fire blight resistance locus on linkage group 10
Source: Mol Breed. 2018 Aug 6;38(8):106. doi: 10.1007/s11032-018-0863-5 (PMC6096517; doi:10.1007/s11032-018-0863-5)
Supplement: Supplementary file 3 — Positions of BAC clones on the Golden Delicious doubled haploid (GDDH13) genome (DOCX 12 kb) [file 11032_2018_863_MOESM3_ESM.docx]

**Table S2**. Positions of BAC clones on the Golden Delicious doubled haploid (GDDH13) genome

| BAC clones | Sequence source | Position on LG10 of GDDH13 | | Orientation |
| --- | --- | --- | --- | --- |
| 36P10 | BAC-ends | 30,279,633 | 30,597,369 | RP-T7 |
| 70N1 | BAC-ends | 30,351,421 | 30,538,626 | RP-T7 |
| 39G5 | BAC-ends | 30,352,191 | 30,615,988 | RP-T7 |
| 62J21 | BAC-ends | 30,412,430 | 30,559,889 | T7-RP |
| 24N24 | BAC-ends | 30,598,166 | 30,814,822 | RP-T7 |
| 46H22 | BAC-ends | 30,598,315 | 30,870,529 | T7-RP |
| 5E10 | BAC-ends | 30,803,214 | 31,009,108 | RP-T7 |
| 95C21 | BAC-ends | 30,995,695 | 31,216,535 | T7-RP |

Golden Delicious Doubled Haploid genome (GDDH13; Daccord *et al*. 2017)
